# High viral load of Merkel cell polyomavirus DNA sequences in Langerhans cell sarcoma tissues

**DOI:** 10.1186/1750-9378-9-15

**Published:** 2014-05-06

**Authors:** Ichiro Murakami, Michiko Matsushita, Takeshi Iwasaki, Satoshi Kuwamoto, Masako Kato, Yasushi Horie, Kazuhiko Hayashi, Jean Gogusev, Francis Jaubert, Shu Nakamoto, Mitsunori Yamakawa, Hirokazu Nakamine, Katsuyoshi Takata, Takashi Oka, Tadashi Yoshino

**Affiliations:** 1 Division of Molecular Pathology, Faculty of Medicine, Tottori University, Yonago 683-8503, Japan; 2 Department of Pathobiological Science and Technology, School of Health Science, Faculty of Medicine, Tottori University, Yonago 683-8503, Japan; 3 Department of Pathology, Tottori University Hospital, Yonago 683-8503, Japan; 4 Inserm U507 and U1016, Institut Cochin, Paris 75014, France; 5 University of Paris Descartes (Paris V), Paris 75006, France; 6 Department of Pathology, Tottori Prefectural Central Hospital, Tottori 680-0901, Japan; 7 Department of Pathological Diagnostics, Yamagata University School of Medicine, Yamagata 990-9585, Japan; 8 Department of Laboratory Medicine, The Japan Baptist Medical Foundation, Kyoto 606-8273, Japan; 9 Department of Pathology, Okayama University Graduate School of Medicine, Dentistry and Pharmaceutical Sciences, Okayama 700-8530, Japan

**Keywords:** Merkel cell polyomavirus, Langerhans cell sarcoma, Langerhans cell, Multiplex quantitative PCR

## Abstract

**Background:**

Langerhans cell (LC) sarcoma (LCS) is a high-grade neoplasm with overtly malignant cytologic features and an LC phenotype. We very recently suggested that LC behaves as a reservoir for common dermotropic Merkel cell polyomavirus (MCPyV) and determined the relationship between LC histiocytosis (LCH), which has an underlining oncogenic capacity, and MCPyV as a trigger for a reactive process rather than a neoplastic process. We propose LC to be a reservoir for MCPyV and hypothesize that some LCS subtypes may be related to the MCPyV agent.

**Findings:**

We examined seven LCS tissues using multiplex quantitative PCR (Q-PCR) and immunohistochemistry with anti MCPyV large-T (LT) antigen antibody. High viral loads of MCPyV DNA sequences (viral load = relative levels of MCPyV) were detected (0.328–0.772 copies/cell (Merkel cell carcinoma (MCC) = 1.0)) using Q-PCR in 43% (3/7) tissues, but LT antigen expression was not observed (0/7).

**Conclusions:**

Frequent MCPyV-DNA amplification suggests that LCS in some patients may be related to MCPyV infection. Moreover, the higher viral load of LCS (median, 0.453 copies/cell) than low load of LCH (0.003, median of 12 cases) (*P* < 0.01) may suggest a virally induced tumorigenic process in some LCS. Although the absence of LT antigen expression may indicate a different role for MCPyV in this pathology, some subtypes of LCS may develop in the background of MCPyV-infected LC. To the best of our knowledge, this is the first report on the relationship between MCPyV and LCS. The recent discovery of MCPyV opened new therapeutic avenues for MCC. These data open novel possibilities for therapeutic interventions against LCS.

## Findings

### Background

Merkel cell polyomavirus (MCPyV) was discovered in 2008 and was linked to the pathogenesis of Merkel cell carcinoma (MCC), which is a rare and aggressive skin cancer occurring in the dermis of individuals aged 60 years or older [1,2]. Approximately 80% MCC harbors MCPyV, indicating its prominent role in the development of the disease. Mechanistically, MCPyV-induced oncogenesis is considered to be induced by MCPyV large T (LT) antigen through molecular binding with the retinoblastoma protein [1]. Several tumorigenic processes leading to MCC were proposed. One was that the induced mutations of MCPyV due to long exposure to ultraviolet light leads to integration of the cytoplasmic viral sequences into the DNA of originating MCC cells. MCPyV primarily resides on the skin, and we have detected MCPyV-DNA in the organs of autopsy cases, with the highest prevalence (53%) in the skin [3].

Because Langerhans cells (LCs) exist above the middle of the spinous zone of epidermis [4] and can capture external pathogens [5] and because of their ability to play roles as antigen-presenting cells [6,7], we proposed that external pathogens may be initially recognized by LC and may subsequently infect Merkel cells which are mostly located at the basal cell layer of the epidermis. Therefore, we hypothesized that LC were a reservoir for MCPyV and demonstrated this phenomenon by showing MCPyV-DNA in microdissected LC of dermatopathic lymphadenopathy (DLA) [8].

In this study, we hypothesized that LC sarcoma (LCS) may originate from a long standing reservoir cell for MCPyV and showed some prevalence of MCPyV-DNA in LCS with high viral load compared with that in non-affected LC.

### Results

#### Quantitative PCR (Q-PCR) for MCPyV-DNA in LCS tissues

The results of Q-PCR tissue analysis for MCPyV are shown in Table 1 with a positive (MCC = 1.0) and negative control (water = −). MCPyV-DNA sequences in three of seven tissues from LCS patients were detected corresponding to high viral loads (0.328–0.772).

**Table 1:** Clinical characteristics and lesional MCPyV data of patients with LCS

**Patient**	**Age (years)**	**Sex**	**Tissue**	Q-PCR (Viral load)*	**MCPyV large T antigen (CM2B4)**
LCS1	81	F	Skin	-	-
LCS2	73	M	LN	-	-
LCS3	72	F	Salivary gland	+ (0.453)	-
LCS4	61	M	Lung	+ (0.772)	-
LCS5	62	M	LN	+ (0.328)	-
LCS6	40	M	LN	-	-
LCS7	81	F	LN	-	-

The median age of the LCS patients (n = 7) was 72 years (range, 42–81 years of age). *Positive control (MCC):+ (Viral load = 1.0), Negative control (double distilled water): −. Viral load, relative levels of MCPyV. *Abbreviations*: *LCS* Langerhans cell sarcoma, *LN* lymph node, *MCC* Merkel cell carcinoma, *MCPyV* Merkel cell polyomavirus, *Q-PCR* multiplex quantitative PCR for MCPyV.

#### Immunohistochemistry for MCPyV-LT antigen

**Figure 1:**
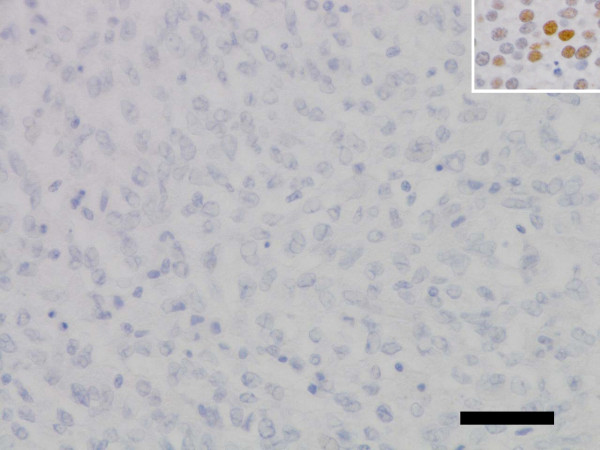
**No immunoreactivity for MCPyV-LT using CM2B4 antibody in LCS tissue lesion (LCS4).** Insert: MCC tissue lesion as a positive control. Scale bar, 50 μm. *Abbreviations*: *CM2B4*, a monoclonal antibody to MCPyV-LT; *LCS*, Langerhans cell sarcoma; *MCC*, Merkel cell carcinoma; *MCPyV*, Merkel cell polyomavirus; *MCPyV-LT*, large T antigen protein of MCPyV.

Neither cytoplasmic nor nuclear immuno-reactivity for this protein was observed in LCS cells as reported in Figure 1 and Table 1. MCC as a positive control is shown in Figure 1.

### Discussion

LC is an epidermal dendritic cell (DC) with antigen-presenting cell capacities. Immature DC typically responds to pathogen exposure by specific maturation processes that facilitate induction of further innate and adaptive immune responses [6,9]. However, some viruses, such as HIV, vaccinia, measles, and dengue, interfere with DC function and maturation to escape the immune surveillance [9-13].

Buffy coats of healthy donors above 20 years of age examined for MCPyV-DNA showed a 22% prevalence [14]. In this study [14] the cellular reservoir was not shown. In another investigation [15], CD14+ activated monocytes in peripheral blood were shown to serve as a reservoir for MCPyV. Recently we have described the prevalence of MCPyV in human tissues from 41 autopsy cases, i.e., skin (53%), lymph node (0%, excluding DLA), and lung (8%), with low viral load (viral load = 0.00026–0.22) [3]. Our data indicated the possibility that MCPyV is a dermotropic virus. We have previously shown the prevalence of MCPyV in LC in DLA tissues (viral load = 0.001–0.006) and in LC histiocytosis (LCH) lesions (viral load = 0.0001–0.033) [8]. Our data using DLA tissue, in which epidermal LCs with MCPyV migrate into lymph nodes [16,17], suggest that LC is a candidate reservoir of MCPyV in the epidermis. MCPyV may also interfere with LC functions similar to some viruses, as mentioned above.

**Figure 2:**
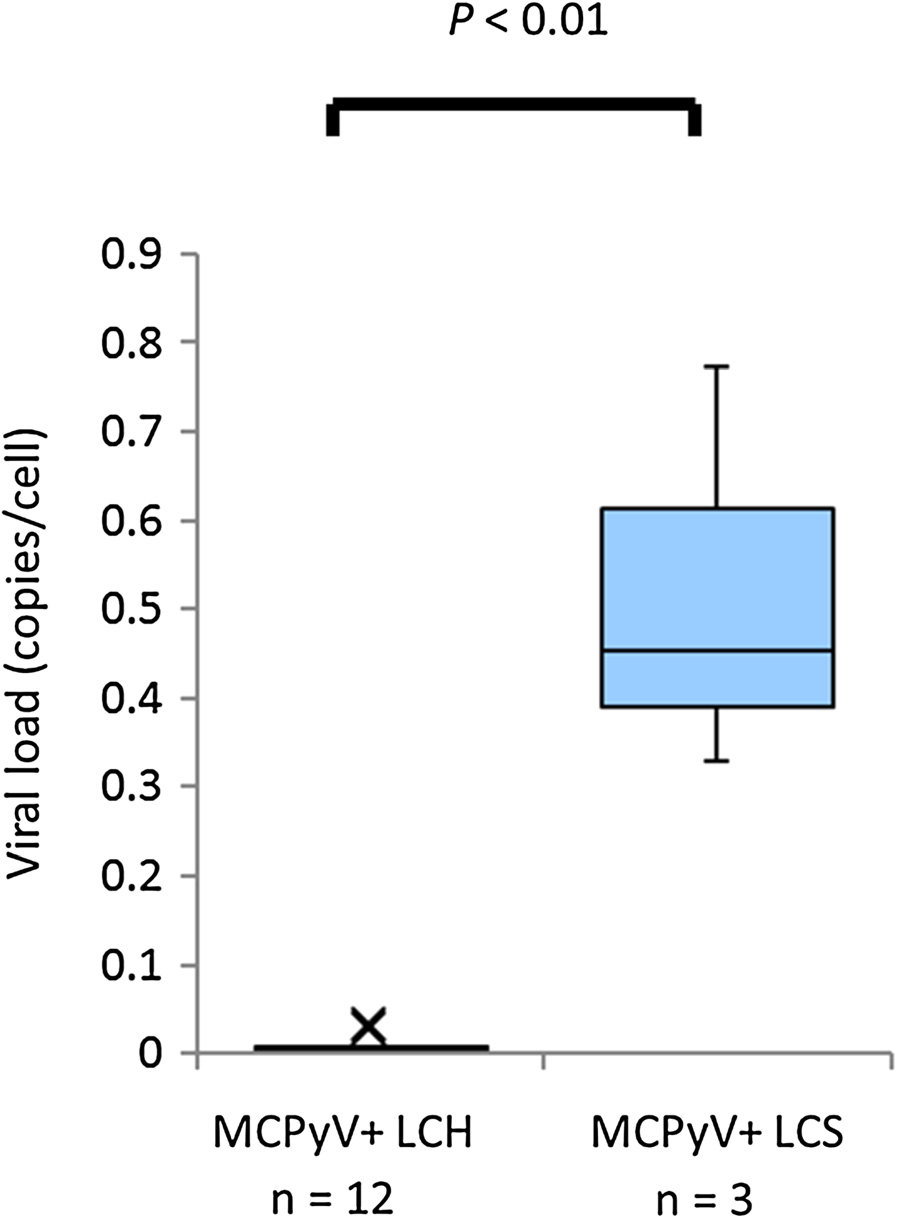
**Q-PCR data comparing MCPyV-positive LCH samples and MCPyV-positive LCS samples.** Q-PCR provides the viral load of MCPyV (relative level of MCPyV (MCC = 1)) for MCPyV-positive LCH samples and MCPyV-positive LCS samples plotted as box-whisker plots (Mann–Whitney *U* test, *P* < 0.01). The median viral load data are 0.003 and 0.453 in MCPyV-positive LCH and MCPyV-positive LCS, respectively. *Abbreviations*: *LCH*, Langerhans cell histiocytosis; *LCS*, Langerhans cell sarcoma; *MCC*, Merkel cell carcinoma; *MCPyV*, Merkel cell polyomavirus; *Q-PCR*, multiplex quantitative PCR.

In this study, we have shown the presence of MCPyV in LCS with high viral loads (Table 1, viral load = 0.328–0.772). MCPyV-positive LCS samples were compared with MCPyV-positive LCH samples [8] in the point of MCPyV viral load in Figure 2 with significant difference (P < 0.01). The presence of MCPyV in LCS lesions suggests three possibilities: a) viral infection as a consequence of LCS development, b) LCS as a bystander, and c) a viral causal agent of LCS. Though MCPyV is typically an asymptomatic infection in adults [18], the presence of MCPyV with high viral loads denies the possibility it is a simple bystander within LCS lesions.

Whether MCPyV-DNA is integrated into the nuclei of LCS cells as an oncogenic virus warrants further investigations, but the high viral loads of MCPyV in LCS lesions may suggest an important oncogenic factor in LCS cells. We are now trying specific PCR as Sastre-Garau et al. did [19]. Immunohistochemical negativity for MCPyV-LT (CM2B4) may indicate a different tumorigenetic mechanism that is involved in MCPyV-LT positive MCC [1,20] or under the limit of detection for MCPyV-LT.

### Conclusion

To the best of our knowledge, this is the first report indicating a relationship between MCPyV infection and LCS. Thus, we suggest that MCPyV may play some role as an oncogenic factor in particular subtypes of LCS. Based on the foregoing, we propose an LCS tumorigenesis model that MCPyV may be a cause of LCS. The recent discovery of MCPyV opened new therapeutic avenues for MCC [21]. Although MCPyV-LT expression was not detected, the origin of some forms of LCS may be MCPyV-infected LC. When confirmed, these findings will open novel possibilities for therapeutic interventions against LCS.

### Methods

#### Patients and tissue samples of LCS

This study was approved by the Institutional Review Board of Faculty of Medicine, Tottori University, Yonago, Japan.

A total of seven tissues from patients with LCS were analyzed. All tissues of LCS were obtained as formalin-fixed paraffin-embedded (FFPE) samples.

#### Confirmation of accurate diagnosis of LCS

Diagnostic accuracy of all collected samples, histological sections and immunohistochemistry for CD1a, S100 protein (S100), and CD207 (langerin) of all specimens was confirmed by two pathologists on the basis of the diagnostic criteria [22].

#### DNA extraction from LCS tissues

DNA was extracted from each FFPE sample using the QIAamp DNA FFPE Tissue Kit and Mini Kit (QIAGEN GmbH, Hilden, Germany).

#### Multiplex quantitative PCR (Q-PCR) for MCPyV detection

Q-PCR was performed in a 10-μl reaction mix containing TaqMan® Copy number reference assay RNase P (Applied Biosystems, Foster City, CA, USA) as internal control [8]. A primer pair targeting the position 859–934 (*MCPyV-LT*) on MCC350 (GenBank EU375803) was [20] used. To determine the MCPyV-DNA ratio relative to MCPyV-DNA of the reference MCC (MCC = 1.0) for each case, Q-PCR was performed using an ABI PRISM 7900HT Sequence Detection System (Applied Biosystems) as previously described [20]. The ratio of the virus was determined using the viral signal in a positive MCC sample as a reference (viral load = relative levels of MCPyV, MCC = 1.0 copy/cell). Thresholds were plotted against each standard sample. All reactions of samples and controls were performed in triplicate, and the average was reported. The MCPyV-DNA ratio in each sample was determined on the basis of corresponding standard curves.

#### Immunohistochemistry for detection of MCPyV-LT antigen

For detection of MCPyV-LT expression, immunohistochemistry was performed using monoclonal antibody CM2B4 (mouse monoclonal IgG2b, 200 μg/ml, sc-136172, Santa Cruz Biotechnology, CA, USA) generated against a peptide fragment of MCPyV-LT as immunogen [23,24].MCC samples were used as controls throughout.

#### Statistical analysis

Comparisons of MCPyV viral load between MCPyV-positive LCH and MCPyV-positive LCS were performed using the Mann–Whitney *U* test. Differences between values were considered statistically significant at *P* < 0.05.

## Abbreviations

DC: Dendritic cell; DLA: Dermatopathic lymphadenopathy; FFPE: Formalin-fixed paraffin-embedded; LC: Langerhans cell; LCH: Langerhans cell histiocytosis; LCS: Langerhans cell sarcoma; LN: lymph node; MCC: Merkel cell carcinoma; MCPyV: Merkel cell polyomavirus; MCPyV-LT: Large T antigen protein of MCPyV; Q-PCR: Multiplex quantitative PCR.

## Competing interests

The authors declare no competing financial interests.

## Authors’ contributions

IM, KH, and HN conceived the initial study proposal. IM, KH, and JG were responsible for writing the manuscript. All authors were involved in the design of the research. MM, TI, and SK were involved in the analysis of the data. All authors have critically reviewed and approved the manuscript.

## References

[B1] Feng H Shuda M Chang Y Moore PS Clonal integration of a polyomavirus in human Merkel cell carcinoma Science 2008 319 1096 1100 10.1126/science.1152586 18202256 PMC2740911

[B2] Kuwamoto S Recent advances in the biology of Merkel cell carcinoma Hum Pathol 2011 42 1063 1077 10.1016/j.humpath.2011.01.020 21641014

[B3] Matsushita M Kuwamoto S Iwasaki T Higaki-Mori H Yashima S Kato M Murakami I Horie Y Kitamura Y Hayashi K Detection of merkel cell polyomavirus in the human tissues from 41 Japanese autopsy cases using polymerase chain reaction Intervirology 2013 56 1 5 10.1159/000338620 22986833

[B4] Kanik A Li M Uramacher CD Mills SE Normal skin Histology for pathologist 2012 Philadelphia: Lippincott Williams & Wilkins 3 28

[B5] Kubo A Nagao K Yokouchi M Sasaki H Amagai M External antigen uptake by Langerhans cells with reorganization of epidermal tight junction barriers J Exp Med 2009 206 2937 2946 10.1084/jem.20091527 19995951 PMC2806471

[B6] Banchereau J Steinman RM Dendritic cells and the control of immunity Nature 1998 392 245 252 10.1038/32588 9521319

[B7] Weitzman S Egeler RM Weitzman S, Egeler RM Histiocytic disorders of children and adults: introduction to the problem, overview, historical perspective and epidemiology Histiocytic disorders of children and adults 2005 Cambridge: Cambridge University Press 1 13

[B8] Murakami I Matsushita M Iwasaki T Kuwamoto S Kato M Horie Y Hayashi K Imamura T Morimoto A Imashuku S Gogusev J Jaubert F Takata K Oka T Yoshino T Merkel cell polyomavirus DNA sequences in peripheral blood and tissues from patients with Langerhans cell histiocytosis Hum Pathol 2014 45 119 126 10.1016/j.humpath.2013.05.028 24321520

[B9] da Costa CET Annels NE Egeler RM Weitzman S, Egeler RM, Cambridge UK The immunological basis of Langerhans cell histiocytosis Histiocytic disorders of children and adults 2005 Cambridge, UK: Cambridge University Press 66 82

[B10] Grosjean I Caux C Bella C Berger I Wild F Banchereau J Kaiserlian D Measles virus infects human dendritic cells and blocks their allostimulatory properties for CD4+ T cells J Exp Med 1997 186 801 812 10.1084/jem.186.6.801 9294135 PMC2199052

[B11] Engelmayer J Larsson M Subklewe M Chahroudi A Cox WI Steinman RM Bhardwaj N Vaccinia virus inhibits the maturation of human dendritic cells: a novel mechanism of immune evasion J Immunol 1999 163 6762 6768 10586075

[B12] Tortorella D Gewurz BE Furman MH Schust DJ Ploegh HL Viral subversion of the immune system Annu Rev Immunol 2000 18 861 926 10.1146/annurev.immunol.18.1.861 10837078

[B13] Izmailova E Bertley FM Huang Q Makori N Miller CJ Young RA Aldovini A HIV-1 Tat reprograms immature dendritic cells to express chemoattractants for activated T cells and macrophages Nat Med 2003 9 191 197 10.1038/nm822 12539042

[B14] Pancaldi C Corazzari V Maniero S Mazzoni E Comar M Martini F Tognon M Merkel cell polyomavirus DNA sequences in the buffy coats of healthy blood donors Blood 2011 117 7099 7101 10.1182/blood-2010-09-310557 21464370

[B15] Mertz KD Junt T Schmid M Pfaltz M Kempf W Inflammatory monocytes are a reservoir for Merkel cell polyomavirus J Invest Dermatol 2010 130 1146 1151 10.1038/jid.2009.392 20016500

[B16] Shamoto M Osada A Shinzato M Kaneko C Yoshida A Do epidermal Langerhans cells, migrating from skin lesions, induce the paracortical hyperplasia of dermatopathic lymphadenopathy? Pathol Int 1996 46 348 354 10.1111/j.1440-1827.1996.tb03620.x 8809881

[B17] O'Malley DP George TI Orazi A Abbondanzo SL King DW Dermatopathic lymphadenitis Atlas of nontumor pathology, First series, Fascicle 7, Benign and reactive conditions of lymph node and spleen 2009 Washington, DC: American registry of pathology 143 145

[B18] Tolstov YL Pastrana DV Feng H Becker JC Jenkins FJ Moschos S Chang Y Buck CB Moore PS Human Merkel cell polyomavirus infection II. MCV is a common human infection that can be detected by conformational capsid epitope immunoassays Int J Cancer 2009 125 1250 1256 10.1002/ijc.24509 19499548 PMC2747737

[B19] Sastre-Garau X Peter M Avril MF Laude H Couturier J Rozenberg F Almeida A Boitier F Carlotti A Couturaud B Dupin N Merkel cell carcinoma of the skin: pathological and molecular evidence for a causative role of MCV in oncogenesis J Pathol 2009 218 48 56 10.1002/path.2532 19291712

[B20] Kuwamoto S Higaki H Kanai K Iwasaki T Sano H Nagata K Kato K Kato M Murakami I Horie Y Yamamoto O Hayashi K Association of Merkel cell polyomavirus infection with morphologic differences in Merkel cell carcinoma Hum Pathol 2011 42 632 640 10.1016/j.humpath.2010.09.011 21277612

[B21] Schrama D Ugurel S Becker JC Merkel cell carcinoma: recent insights and new treatment options Curr Opin Oncol 2012 24 141 149 10.1097/CCO.0b013e32834fc9fe 22234254

[B22] Jaffe R Weiss LM Facchetti F Swerdlow SH, Campo E, Harris NL, Jaffe ES, Pileri SA, Stein H, Thiele J, Vardiman JW Tumours derived from Langerhans cells WHO Classification of Tumours of Haematopoietic and Lymphoid Tissues 2008 Lyon, France: IARC 358 360

[B23] Busam KJ Jungbluth AA Rekthman N Coit D Pulitzer M Bini J Arora R Hanson NC Tassello JA Frosina D Moore P Chang Y Merkel cell polyomavirus expression in merkel cell carcinomas and its absence in combined tumors and pulmonary neuroendocrine carcinomas Am J Surg Pathol 2009 33 1378 1385 10.1097/PAS.0b013e3181aa30a5 19609205 PMC2932664

[B24] Shuda M Arora R Kwun HJ Feng H Sarid R Fernandez-Figueras MT Tolstov Y Gjoerup O Mansukhani MM Swerdlow SH Chaudhary PM Kirkwood JM Nalesnik MA Kant JA Weiss LM Moore PS Chang Y Human Merkel cell polyomavirus infection I. MCV T antigen expression in Merkel cell carcinoma, lymphoid tissues and lymphoid tumors Int J Cancer 2009 125 1243 1249 10.1002/ijc.24510 19499546 PMC6388400

